# Primary investigation of rice-fish co-culture: Investigating the feasibility of raising fish in flooded rice paddies in the Southern USA

**DOI:** 10.1371/journal.pone.0327572

**Published:** 2025-07-10

**Authors:** Yulin Jia, Aaron K. Jackson, Bartholomew Green, S. Adam Fuller, Heather Box, LaDuska Sells, Candis Ray, Paxton Harper, Brennan Smith

**Affiliations:** 1 USDA- ARS Dale Bumpers National Rice Research Center (DB NRRC), United States of America; 2 USDA- ARS Harry K Dupree Stuttgart National Aquaculture Research Center (SNARC), United States of America; 3 USDA-ARS Food Processing and Sensory Quality Research Group, New Orleans, Louisiana, United States of America; Bangladesh Agricultural University, BANGLADESH

## Abstract

With limited land and water resources available it has become critical to maximize output for agricultural products while maintaining sustainable farming practices. Rice-fish co-culture can be used to maximize limited land and water resources for production of rice and fish concurrently and reduce the need of chemical inputs. While rice-fish co-culture has been practiced in Asia for centuries, very few studies have examined the feasibility and economic impact of rice fish co-culture in the U.S.A. Two studies were conducted in Stuttgart, Arkansas, U.S.A. from 2023-2024 to investigate the suitability of raising Koi carp in flooded rice paddies with a sushi rice variety, Eclipse. The objectives of the studies were to determine if co-cultivation of Koi carp and rice in the Southern U.S.A. could enhance rice production and quality while providing additional economic return from Koi carp. Our studies showed that Koi carp with two different initial weights can be grown concurrently in a flooded rice-fish production system and result in enhanced rice yield, milling yield, and protein content, and positive estimated net returns for Koi carp production. This system appears to offer U.S.A. rice farmers a potential diversification strategy for smaller fields where specialty rice varieties are grown, however, a full economic feasibility study still is needed.

## 1. Introduction

Rapid population growth can limit land and water characteristics that are directly related to agricultural production which in turn may increase malnutrition and lead to food quality and shortage issues [1]. Rice is the main source of carbohydrates grown in Asia [2], and its production is a major consideration for worldwide food security [3]. The production of both rice and fish independently requires substantial land and water resources. Growing fish in rice paddies is an ancient farming practice in several rice-growing areas in Asia including provinces in South and Southwest China, which can be dated back to over twenty centuries ago [4,5]. Prior to the 1970s, the focus of rice-fish culture was to grow rice while the fish provided animal protein primarily for consumption by the farmer. As access to both local and global markets increased many farmers began to realize fish production could be economically profitable and provide income equal to or even greater than their rice crop. By the end of the 1990s, the area of rice-fish farming in China reached 1.464 million ha, which further expanded to 1.528 million ha in 2001. Food fish production from paddy fields increased from 206,900 tons in 1994 by 214% to 649,996 tons in 1999, with a further increase to 849,055 tons by 2001. The increase in food fish production from rice paddies was accompanied with gains in overall rice yield averaging 225 kg per ha between 1994–1999 [6].

The production of rice resulting in increased quality and quantity while reducing pesticide and fertilizer use has been investigated under various sustainable and economic conditions [7]. The integration of rice with aquatic animals such as ducks, fish, mussels, shrimp, and crab are referred to as the integrated agri-aquaculture farming system (IAAFS). The goal of IAAFS is to promote sustainable agri-aquaculture development [8]. In Asia the culturing of loach (*Misgurnus anguillicaudatus*), tilapia (*Oreochromis* sp.), catfish (*Clarias* sp.), shrimp (*Macrobrachium rosenbergii*), and common carp (*Cyprinus carpio*) have been found to be most suitable in rice paddies [9]. The system decreases environmental pollution and improves water and land resource usage while providing grain and meat for consumers [10].

In the southern U.S.A., particularly in Louisiana, co-cultivation of rice and crawfish (*Procambarus clarkii*) has been successful for many years. In 2017, 33.34% of the rice acreage of Louisiana used rice-crawfish co-cultivation methods, and current estimates as of 2024 list roughly 72,843 hectares in use for rice-crawfish co-cultivation [11,12]. In many instances, a sequential rotation is used in which crawfish are added to a rice paddy when the rice is tall enough to shade the water. The crawfish burrow into the mud during dry down and harvest of the rice; then, paddies are reflooded and the crawfish are harvested early the following spring. The benefits of rice-crawfish co-cultivation techniques include decreased disease instances of sheath blight, reduced prevalence of weedy red rice, and additional monetary income from crawfish ranging from $324–486 per hectare [11]. Despite the success of rice-crawfish co-cultivation, few studies in the U.S.A have examined rice-fish co-cultivation techniques.

In recent decades, the aquaculture sector has garnered significant interest due to maximizing production of aquatic food. Rice-fish co-culture was shown to reduce insect pest populations and lower the requirement for agro-chemical usage including pesticides and herbicides while increasing income of the farmer [13]. Development of rice-fish culture in Arkansas, U.S.A., began with the enactment of Public Law 85–342 to establish an experiment station and research program that included rice-fish culture [14]. The Fish Farming Experimental Laboratory was established at Stuttgart, Arkansas, beginning in 1960 and later became the Harry K Dupree Stuttgart National Aquaculture Research Center [15]. A survey of Arkansas farmers in 1958 who used rice-fish rotation described problems encountered in adopting fish culture, reported rice yields in rotation with fish culture of 38–51 bushels/hectare without fertilizer treatment compared to an average 31 bushels/hectare in fertilized fields without fish, and detailed positive economic returns for the various combinations of fish cultured [16]. However, a subsequent economic evaluation of the rice-fish rotation revealed less favorable returns compared to rice-soybean or rice-fallow rotation schemes [17]. More recently, rice-fish sequential rotation is being investigated in an ongoing on-farm experiment in eastern Arkansas to improve sustainability of existing rice farming practices [18]. However, concurrent rice-fish culture has not been commercially implemented in Arkansas or any state in the U.S.A.

Characteristics of fish suitable for concurrent rice-fish culture include benthopelagic food habits, hardiness, tolerance of shallow water depths, variable and high-water temperatures, low dissolved oxygen concentrations, and availability of fingerlings of the desired size with good market value. Among the variety of fish species grown traditionally in rice-fish culture in Asia, common carp is a preferred species [19,20]. A candidate fish for concurrent rice-fish culture in the U.S. should, in addition to the above-referenced characteristics, attain a marketable size within the 120–150-day rice growing period. One such candidate is the Koi carp, an ornamental variant of common carp that varies widely in coloration and is a preferred fish for water gardens in the southern U.S.A. [21–23]. Taxonomy of Koi Carp is somewhat ambiguous due to its many years of domestication with most classifying them as *Cyprinus carpio* L. of various subspecies. Koi carp are benthivorous fish that feed on insects, benthic organisms, plant material, algae, seeds, organic matter, and crustaceans [24–26].

Rice and fish both require sufficient water resources, which may be in limited supply for many areas, to be profitable. The implementation of sustainable farming practices doesn’t necessarily lead to trade-offs in profit or yield. Successful co-culturing practices can save water; reduce fuel inputs for pumping water; reduce pesticide, herbicide and fertilizer use; increase yields; and expand farm products that are economically favorable [8,27]. To assess the suitability of rice-fish co-culture in the southern U.S.A. we conducted two, concurrent rice-Koi culture studies in consecutive years with the objective to evaluate its effects on production, milling quality, yield, and protein content of rice, while examining the possibility of additional economic return from fish production.

## 2. Materials and Methods

### 2.1. Experiment design

**Fig 1:**
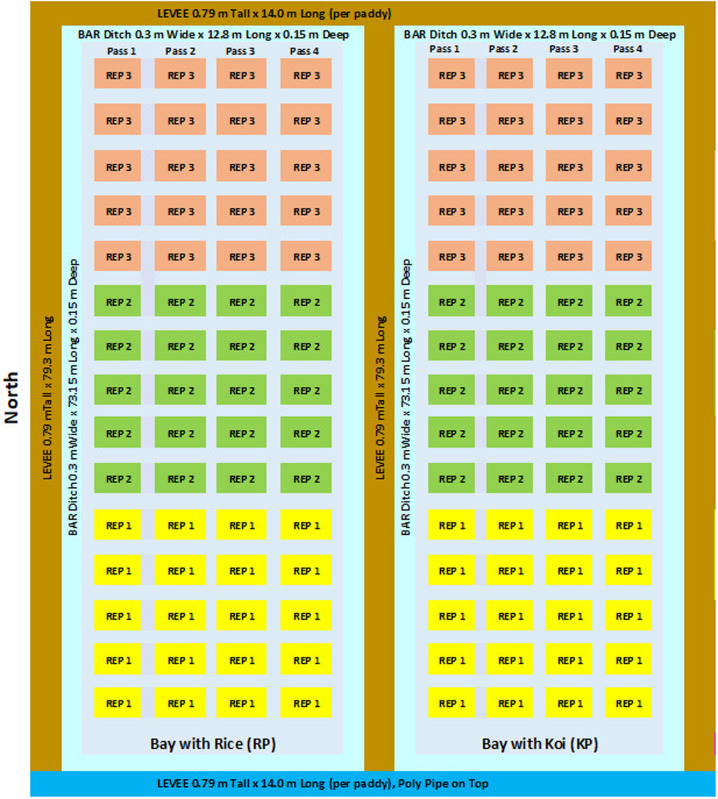
Paddy layout for Study 1.

**Fig 2:**
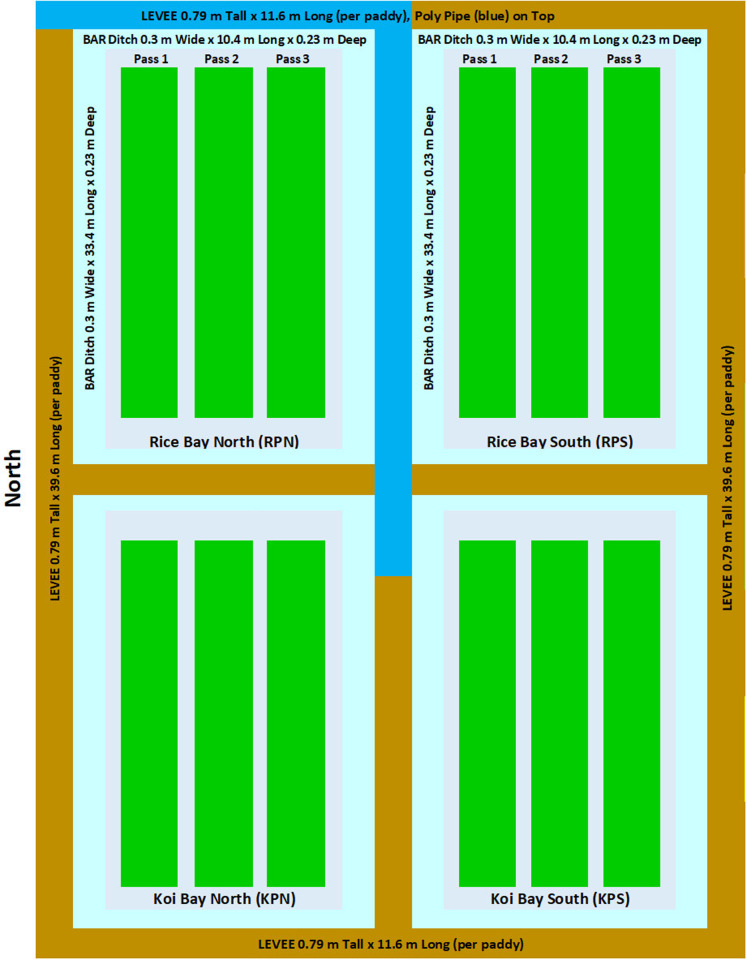
Paddy layout for Study 2.

These studies were conducted at the Dale Bumpers National Rice Research Center (DB NRRC), Agricultural Research Service (ARS), U.S. Department of Agriculture, Stuttgart, Arkansas, U.S.A. The soil at Stuttgart, Arkansas is characterized as a Dewitt silt loam. Two contiguous 0.111-ha paddies were used for 2023 (Study 1), and four contiguous 0.042-ha paddies were used for 2024 (Study 2). Each paddy in Study 1was 42.06 m wide x 73.15 m long with a 0.3-m wide x 0.15-m deep bar ditch and 0.79-m tall levees (Fig 1). One paddy was planted only with rice (RP), the other a rice-Koi co-culture (KP). In Study 2, each paddy was 11.58 m wide x 39.62 m long, the bar ditch was 0.3 m wide x 0.23 m deep, and the levee was 0.79 m tall (Fig 2). Rice only paddies were labeled RPN (rice paddy north) and RPS (rice paddy south), while rice-Koi paddies were labeled KPN (Koi paddy north) and KPS (Koi paddy south).

Study 1 paddies were planted on 4/17/2023, as a randomized complete block design with three replications each containing twenty 6-row plots (1.83-m wide x.3.05-m long; 30-cm row spacing) per replicate. In Study 2, paddies were planted on 4/16/2024 as a randomized complete block design with two replicates per treatment (Koi presence/absence). Each paddy was bulk-planted as three 5-row continuous strips (1.83 m wide x 33.53 m long; 38-cm row spacing). Seed of the rice variety Eclipse (sourced from the 2018/DB NRRC Eclipse increase) was planted in both studies using a Hege planter (Hege Equipment Inc. Colwich, Kansas, U.S.A. planter model Hege 1000 series Drill). Seeding rate for each Study 1 plot was 112 kg/ha and for each Study 2 strip was 120 kg/ha. Agronomic data for each study is shown in Table 1. No further herbicide applications were made to paddies in either study after fish were stocked.

**Table 1:** Agronomic data relative to planting date^1^ for Studies 1 and 2.

	Day	
	Study 1	Study 2
Fertilize^2^ 67 kg P_2_O_5_/ha as triple superphosphate	− 2	− 1
Fertilize^2^ 101 kg K_2_O/ha as sulfate of potash	− 2	− 1
Fertilize^2^ 11 kg Zn/ha as zinc sulfate	N/A	− 1
Planting	1	1
Herbicide^3^ application: Command 3 ME^®^ 1.17 L/ha	N/A	2
Herbicide^3^ application: Facet L^®^ 1.61 L/ha	N/A	2
Flush irrigated	3	3
Herbicide^3^ application: Prowl H2O^®^ 2.46 L/ha	N/A	9
Herbicide^3^ application: Bolero 8 EC^®^ 3.51 L/ha	N/A	9
Herbicide^3^ application: Propanil 480 EC^®^ 7.02 L/ha	N/A	13
Herbicide^3^ application: Facet L^®^ 1.53 L/ha	N/A	13
Herbicide^3^ application: Permit^®^ 74 mL/ha	N/A	13
Herbicide^3^ application: Roundup^®^ 2.34 L/ha	17	N/A
Emergence	27	14
Fertilize^2^: 146 kg N/ha as urea	50	48
Paddies flooded	50	49
Koi carp stocked	65	73
Harvest	203	216

^1^Planting (seeding) date was 11 May 2023 (Study 1) and 16 April 2024 (Study 2).

^2^Fertilizer applications based on soil test results.

^3^Mention of commercial products and tradenames in this publication is solely for the purpose of providing detailed descriptions of methods used herein and does not imply recommendation or endorsement by the United States Department of Agriculture. Chemical composition of trademarked herbicides is provided in S1 Table.

### 2.2. Rice agronomic traits

#### Panicle traits.

In 2023, ten panicles were collected from each of 12 individual plots of both the RP and KP bays. Panicle length, the number of primary branches per panicle, panicle weight, and the number of kernels per panicle were measured for each panicle. Panicle length was measured as the distance between the neck-panicle node to the panicle tip in centimeters. The number of primary branches per panicle and number of kernels per panicle were manually counted. Panicle weight was the total weight of the panicle in grams. Thousand grain weight was calculated from the rough kernel weight (g) of 100 seeds from one panicle per plot and then multiplied by 10.

In 2024, eight panicles were collected from each of three subplots in RPS, RPN, KPS, and KPN. Panicle traits were measured as described above; however, the number of kernels per panicle was not collected for 2024, and thousand grain weight was collected from a single panicle per subplot.

#### Milling and yield.

Milling and yield measurements were collected from the RPN, RPS, KPN, and KPS bays in 2024. Twenty-five panicles were collected from each of the four bays on 3 dates (8/18/2024, 10/18/2024 and 10/30/2024) to calculate total rice (milled whole kernels plus broken kernels), whole milled rice (milled unbroken kernels), and the percent milling yield. Whole milled rice was obtained from rough rice mechanically dehulled using a laboratory rice miller (PAZ-1 DTA, Zaccaria, Limeria, Brazil). The percent milled rice was calculated as whole milled rice divided by the total rice x 100. To estimate overall yield, a 10-meter row in each bay was hand harvested on 10/30/2024 and threshed in the field with a portable thresher (SBT, Almaco, Nevada, Iowa,U.S.A.). The dirty weight (not cleaned of chaff) of the rough rice was extrapolated to kg/ha.

#### Protein content.

Milled rice was ground in an electric coffee grinder (Krups F20342, Mexico) for protein quantitation. The percent protein of rice samples was determined by the Dumas nitrogen combustion method according to AOAC method 992.23 (AOAC, 1995) with a Thermo Scientific FlashSmart EA (CE Elantech, Lakewood, New Jersey, U.S.A.) with tin foil cups (Thermo Fisher Scientific, part no. 252–08000) used to contain samples. A 6.25 nitrogen conversion factor was used to convert the percent nitrogen into percent protein.

### 2.3. Experiment design with stocking rate

Rice-fish culture has a long history in Asia for which a base of literature exists. Additionally, carp aquaculture in (eastern) Europe has been practiced for hundreds of years and ample stocking/yield information exists for carp culture in Europe. About 6,000 fish/ha are stocked into paddies in rice-fish culture systems, fed a supplemental feed daily, with reported fish yields of 850–1,200 kg/ha [28–30]. Chemical fertilizer will be the only nutrient input used in both studies and will be added prior to flooding the rice paddies. The fish will rely on natural productivity (e.g., insects, benthic organisms, etc.) for nutrition; this level of nutrient input is classified as extensive management. Expected fish yield ranges from 400-600 kg/ha in extensively managed systems [28,29]. For Study 1 expected gross fish yield and final individual weight were estimated conservatively at 400 kg/ha and 100 g/fish, respectively, when 15-g average weight fish were stocked. Based on Study 1 results expected Study 2 gross fish yield and final individual weight were estimated at 600 kg/ha and 300 g/fish, respectively, when 150-g average weight fish were stocked. Stocking rate for each study was calculated based on these estimates.

Fingerling Koi carp (14.9 ± 2.1 g/fish; mean ± SE) were stocked for the first study in one paddy (KP) at 4,493 fish/ha on June 13, 2023, while the other paddy had no fish. Fish were harvested on 10/12/2023, 121 days after stocking. Fish harvested from the first study were overwintered in indoor tanks. In preparation for stocking the second study fish were separated manually into two equal-numbered lots; to minimize handling stress, fish were separated visually to achieve populations with similar size distributions. On 6/18/2024, two paddies (KPN and KPS) each were stocked with 110 Koi carp of average weight 275.3 ± 94.2 g (KPN) and 278.1 ± 117.2.0 g (KPS) with 2595 fish/ha in both KPN and KPS. Fish were harvested on 10/21/2024, 125 days after stocking. Fish were not fed any supplemental feed during either experiment.

For both studies, each paddy was drained to harvest the fish. A net cage was suspended at the paddy outlet to capture fish. Additionally, a 3-m long seine and handheld dipnets were used to harvest fish from the peripheral levee bar ditch. Any fish that escaped capture were collected by hand as soon as the paddy was fully drained. Individual weight (to the nearest 0.1 g) was measured on 103 fish from Study 1 and from all fish from each paddy in Study 2. Remaining fish from KP in Study 1 were weighed in bulk (ca. 25 fish/lot). Consultation with a local Koi carp farmer revealed a Koi wholesale price of $44.092/kg independent of fish size (Fish farmer, personal communication, 11/8/2024). Cost of stocked fish and value of harvested fish were based on this price.

### 2.4. Water parameter measurements

Dissolved oxygen (DO), temperature, and pH in paddy water were measured in the morning and afternoon (0700–0800 and 1300–1500 hours) at 4-day intervals using a handheld meter (HQ series HQ2200 meter, Hach Company, Loveland, Colorado, U.S.A.) during both studies. Water samples from two locations within each paddy were collected (0700–0800 hours) at 8- and 12-d intervals during Study 1 and Study 2, respectively, and transported to the laboratory for immediate analysis. In the lab, sample pH was measured electrometrically. After filtering water through a 0.2-μm membrane filter, water was analyzed for nitrite‑nitrogen (NO_2_-N, diazotization), nitrate‑nitrogen (NO_3_-N, cadmium reduction), and soluble reactive phosphorus (SRP; PO_4_-P, ascorbic acid method) using flow injection analysis according to manufacturer instructions (FIAlyzer 2000; FIAlab Instruments, Seattle, Washington, U.S.A.). Total ammonia‑nitrogen (TAN, NH_3_-N) was analyzed fluorometrically using the flow-injection methodology of Genfa and Dasgupta (1989) [31]. Phytoplankton biomass in unfiltered samples was estimated by chlorophyll *a* analysis according to Lloyd and Tucker (1988) [32]. Total suspended solids (TSS) in unfiltered samples were measured using methods given by Eaton et al., (2005) [33]. Any dead or moribund fish encountered during the twice daily (morning and afternoon) observations were removed, weighed, and the data recorded.

### 2.5. Statistical analysis

As there were differences in growth conditions and experimental design, all rice trait data was examined independently for each year using a mixed model with a restricted maximum likelihood (REML) method to generate best linear unbiased estimate (BLUEs) in JMP (18.1.2) [34]. BLUEs were used rather than averages to account for any variance present within plots, subplots, or reps. All rice tested was the same cultivar, Eclipse.

#### Panicle traits.

In 2023 BLUEs for panicle related traits were generated using treatment (fish versus no-fish control bay) as a fixed effect with plot and rep (panicle) as random effects. In 2024, an additional bay each for fish and no-fish control was added. A similar mixed model approach was used for 2024 panicle traits with treatment serving as the fixed effect and plot, subplot, and rep as random effects. Panicle traits collected in 2023 included panicle length, primary branches, number of kernels, panicle weight, and thousand grain weight. The same panicle traits, excluding number of kernels per panicle, were collected in 2024. In both 2023 and 2024 one rep was collected from each plot or subplot respectively to measure thousand grain weight. A mixed model was applied to obtain BLUEs for thousand grain weight using treatment as the fixed effect and plot or subplot as the random effect for 2023 and 2024, respectively.

#### Milling and yield.

In 2024, milling data was collected across three harvest dates on 8/18/2024, 10/18/2024, and 10/30/2024. Data from the separate harvest dates were combined to constitute a single yearly harvest date. BLUEs were generated using a mixed model with REML in JMP (18.1.2) [34] with treatment as the fixed effect and plot as the random effect. A single measurement from each plot in 2024 was used to calculate the dirty weight (chaff and rough rice) of rice harvested which was used to extrapolate the yield in kg/ha. Student’s t tests were run on all traits to determine significance.

#### Protein content.

The percent protein was calculated in 2024. Two reps consisting of five panicles from different plants in each plot pooled together were collected across three harvest dates (8/18/2024, 10/18/2024, and 10/30/2024). Eighteen grams of bulked rough rice was sent from each treatment from the three different dates for protein analysis. The separate harvest dates were combined as a single harvest date for data analysis. BLUEs were generated using a mixed model with REML in JMP (18.1.2) [34] with treatment as the fixed effect, plot as a random effect, and rep nested within plot as a random effect.

#### Water parameter measurements.

For all water sample data homoscedasticity (Levine’s test) and normality (Shapiro-Wilk test) were evaluated in SAS. Then, water sample results were analyzed using PROC TTEST (SAS version 9.4, SAS Institute Inc., Cary, North Carolina, U.S.A.) [35]. Differences detected in Student’s t test were declared significant at *p *≤ 0.05.

## 3. Results

### 3.1. Fish gain with economic prediction

Fish production results are shown in Table 2. Averaging the results of KPN and KPS together for Study 2, individual Koi carp, gained 92.9 g/fish and 134.2 g/fish during Studies 1 and 2 respectively, equivalent to gains of 623% and 48%. Net fish yield from Study 1 was 24.5% higher than from Study 2. On the other hand, survival of fish in Study 1 was 31.5% lower than for Study 2. The higher net fish yield in Study 1 was attributed to the low initial biomass (67 kg/ha) with stocking a 14.9-g average weight fish compared to the Study 2 higher initial biomass (718 kg/ha) with stocking a 276.7g average weight fish. Although the nature and scope of these experiments precluded formal economic analysis, the estimated cost of fish per hectare, at a wholesale price of $44.092/kg of live Koi carp, for Study 2 was almost 10.7 times more expensive than for Study 1. Although positive in both studies, the estimated net returns were 1.24 times higher in Study 1 ($9,264/ha) compared to Study 2 ($7442/ha average of KPN + KPS). Smaller fish were used in Study 1, which likely contributed to a faster growth rate and reduced survival due to predation.

**Table 2:** Koi carp production data from Studies 1 (2023) and 2 (2024).

Variable	Study 1	Study 2	
	KP	KPN	KPS
Area (ha)	0.111	0.042	0.042
Stock Rate (fish/ha)	4,493	2,595	2,595
Initial Weight (g/fish)	14.9	275.3	278.1
Initial Biomass (kg/ha)	67.2	714.8	722.1
Final Weight (g/fish)	107.8	398.5	423.2
Gain (%)	722	45	52
Gross Fish Yield (kg/ha)	277.3	865.1	908.6
Net Fish Yield (kg/ha)	210.1	150.6	186.8
Survival %	57.0	83.6	82.7
Initial fish cost^1^ ($/ha)	2,963	31,515	31,838
Harvested fish value ($/ha)	12,227	38,160	40,078

^1^ Koi wholesale price $44.092/kg independent of fish size (fish farmer, personal communication, 8 November 2024).

Average morning dissolved oxygen concentration was generally higher in Koi paddies (Table 3). Mean water temperature and pH were within acceptable ranges for Koi carp. Mean daily DO concentrations were 4.67 and 5.21 mg/L and 7.61 and 6.44 mg/L in rice and rice-koi paddies during Studies 1 and 2, respectively. Minimum morning DO concentration ≤ 2.0 mg/L and ≤ 1.0 mg/L occurred on 34.3% and 5.7% of sample dates, respectively, during Study 1, and on 19.4% and 0% of sample dates, respectively, during Study 2 (data not shown). Dissolved oxygen concentrations ≤ 1.0 mg/L in Study 1 KP treatment occurred between weeks 4–6. Prolonged exposure to severe hypoxia (< 2.0 mg/L dissolved oxygen concentration) may result in fish mortality.

**Table 3:** Mean (range) morning and afternoon dissolved oxygen concentration, temperature, and pH of rice paddy water during Studies 1 and 2.

Variable	Study 1		Study 2	
	Rice Paddy	Rice-Koi Paddy	Rice Paddies	Rice-Koi Paddies
MORNING				
Dissolved Oxygen (mg/L)	1.86 (0.58–5.49)	2.58 (0.58–6.99)	2.25 (0.48–7.13)	3.14 (1.70–7.82)
Water Temperature (°C)	24.7 (16.8–30.1)	25.1 (16.6–30.0)	23.7 (11.1–29.7)	23.6 (12.3–29.5)
pH	7.15 (6.66–7.59)	7.18 (6.74–7.48)	7.20 (6.94–7.56)	7.23 (6.67–7.65)
AFTERNOON				
Dissolved Oxygen (mg/L)	7.47 (2.19–11.92)	7.83 (1.8–15.85)	12.98 (7.17–18.32)	9.74 (5.19–17.96)
Water Temperature (°C)	30.0 (21.3–37.3)	30.1 (20.8–36.3)	27.7 (15.0–36.4)	29.4 (16.3–37.0)
pH	7.46 (6.29–8.83)	7.36 (6.48–8.89)	7.64 (7.07–9.29)	7.65 (7.02–9.58)

Post hoc Student’s t test was run, no significant differences (*p *≤ 0.05) were identified for means within years. Study 1 occurred in 2023, Study 2 occurred in 2024.

All mean water characteristic variable concentrations in both studies were within acceptable ranges for Koi carp (Table 4). The only significant difference was detected in Study 2, where mean TAN concentration was significantly lower in rice-Koi paddies compared to rice paddies. The exact cause for the difference in TAN concentrations is unknown. However, chlorophyll *a* concentration in RPS and RPN paddies in August-September 2024 went through 1−2 boom and bust cycles, where concentrations increased from < 100 to ca. 400 mg/m3 and declined to < 100 mg/m3 and likely was responsible for the higher observed TAN concentrations. Chlorophyll *a* is an indicator of phytoplankton biomass. Phytoplankton are primary consumers of ammonia-nitrogen during rapid growth and are primary sources of ammonia-nitrogen during decomposition following a die off. On the other hand, chlorophyll *a* concentration in KPS and KPN paddies were less variable and < 100 mg/m3 from late July through experiment harvest. The lower chlorophyll *a* concentration in KPS and KPN paddies likely were affected by the increased soil turbidity (higher TSS concentrations) caused by Koi foraging activities. Mean TSS concentrations in rice-Koi paddies in both studies were 3.0-3.5 times higher than in rice paddies without Koi.

**Table 4:** Mean (range) water characteristic variable concentrations in rice paddies during Studies 1 and 2.

Variable	Study 1		Study 2*	
	Rice Paddy	Rice-Koi Paddy	Rice Paddies	Rice-Koi Paddies
Total Ammonia-Nitrogen (mg/L)	0.004 (0–0.033)	0.002 (0–0.022)	0.021 b (0.009–0.037)	0.015 a (0.005–0.030)
Nitrite-Nitrogen (mg/L)	0.000 (0–0.004)	0.001 (0–0.006)	0.005 (0–0.029)	0.003 (0–0.010)
Nitrate-Nitrogen (mg/L)	0.013 (0–0.183)	0.002 (0–0.017)	0.007 (0–0.054)	0.007 (0–0.059)
Soluble Reactive Phosphorus (mg/L)	0.035 (0–0.850)	0.002 (0–0.030)	0.017 (0–0.180)	0.012 (0–0.020)
Total Alkalinity (mg/L as CaCO_3_)	99.7 (44.9–139.9)	152.1 (68.7–161.1)	67.7 –	77.6 –
Chlorophyll *a* (mg/m^3^)	18.62 (0–102.82)	21.19 (0–92.86)	53.16 (3.63–375.34)	43.29 (0.90–194.04)
Total Suspended Solids (mg/L)	15.64 (1.83–124.75)	46.30 (4.07–212.83)	16.62 (2.04–75.58)	57.31 (4.81–186.96)

* Post hoc Student’s t test was run, means within year followed by different letters differed significantly (*p *≤ 0.05). Significant differences were only detected in total ammonia-nitrogen in Study 2, 2024. No significant differences were detected in Study 1, 2023.

### 3.2. Rice yield components, yield and nutritional quality

Rice performed poorly in 2023 due to barnyard grass; therefore, data for yield was not collected. Rice panicle traits and thousand grain weight were examined in 2023 and 2024. Due to differences in growth conditions and plot design, traits were analyzed independently for each year. The only significant difference in panicle traits and thousand grain weight occurred with a small, increase in primary panicle branches for the rice control plot in 2023, but no significant differences were detected in 2024. It’s possible the increased number of weeds present in 2023 may have contributed to environmental factors that influenced primary panicle branch number or that the higher calcium carbonate levels in the rice-fish paddies in 2023 influenced nutrient uptake. One additional panicle trait was recorded in 2023, number of kernels per panicle, which was not measured in 2024. However, there was no significant difference between the fish and no-fish control plots (Table 5).

**Table 5:** Panicle traits and thousand grain weight of rice grown with and without fish.

Trait	Rice + Fish bay(s)*	Standard Error	Rice only bay(s)*	Standard Error
Panicle Length (cm) 2023	18.85 A	0.22	18.79 A	0.22
Panicle Length (cm) 2024	16.42 a	0.78	17.54 a	0.18
Primary Panicle Branches 2023	12.11 A	0.16	11.41 B	0.16
Primary Panicle Branches 2024	9.89 a	0.36	11.04 a	0.35
Panicle Weight (g) 2023	2.59 A	0.09	2.56 A	0.09
Panicle Weight (g) 2024	1.81 a	0.11	2.02 a	0.11
Thousand Grain Weight (g) 2023	26.83 A	0.32	27.83 A	0.34
Thousand Grain Weight (g) 2024	26.33 a	0.43	25.17 a	0.43
Number of Kernels per Panicle 2023	94.87 A	3.02	91.42 A	3.03

All values shown are BLUEs and were measured in 2023 or 2024 at Stuttgart, Arkansas, U.S.A. in either bays with both rice and fish or with rice alone. Years were analyzed independently due to variations in plot design between years.

* Student’s t tests were used to test for significance (*p *≤ 0.05). Treatments not sharing the same letter within a trait category are significantly different, capital letters designate 2023 and lower-case letters 2024.

The amounts of total rice and whole milled rice were analyzed over three harvest dates (9/18/2024, 10/18/2024, and 10/30/2024) during the 2024 growing season. The amount of total rice decreased as the growing season progressed (data not shown) and data from all three harvest dates were analyzed together for comparison of rice grown with or without fish. Total rice was significantly higher by 7.7% in bays with both rice and fish. Whole milled rice was also significantly higher by roughly 68% in rice-fish bays. Likewise, the percent milling yield was also significantly higher in rice-fish bays and revealed a 58% increase in milling yield (Table 6) compared to bays with rice alone. Yield (kg/ha) was based on the dirty weight (rough rice without chaff removed) harvest of a 10 m row collected on 10/30/2024. Bays with both rice and fish had over a 129% yield increase compared to bays with rice alone (Table 6). Protein was analyzed in 2024 and rice grain from bays with fish and rice was significantly higher in protein by 22.6% than bays with rice alone (Table 6)

**Table 6:** Yield, milling yield and protein content of rice grown with and without fish.

Trait	Rice + Fish bays*	Standard Error	Rice only bays*	Standard Error
Total Rice (g)	68.33 A	0.57	63.42 B	0.57
Whole Milled Rice (g)	28.67 A	1.42	17.05 B	1.42
Milling Yield (%)	41.56 A	2.34	26.29 B	2.34
Yield (kg/ha)	6130.5 A	298.6	2675.4 B	298.6
Protein (%)	9.32 A	0.12	7.60 B	0.12

Values shown for total rice, whole milled rice, percent milling yield, and percent protein are BLUEs measured in 2024 in Stuttgart, Arkansas, U.S.A. in 2 bays each of both rice and fish or with rice alone. Yield was estimated from the dirty weight (rough rice without chaff removed) of a 10 m row in each plot and converted into kg/ha.

*Student’s t test was used to test for significance (*p *≤ 0.05), values not sharing the same letter within a trait category are significantly different.

### 3.3. Weed control

**Fig 3:**
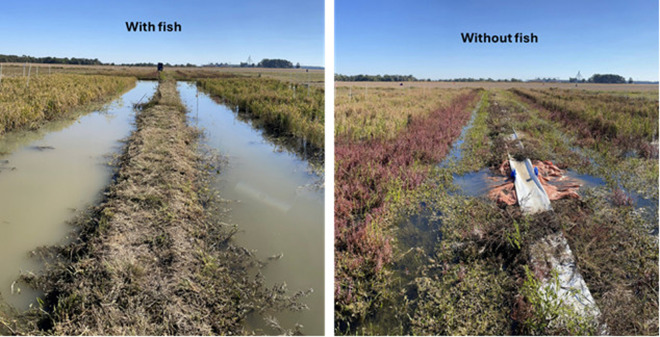
Photos were taken in October 2024 before harvesting Study 2 and show a substantial reduction of weeds in rice paddies with fish versus rice alone.

Weed control was not measured as a quantitative trait, but visually there was a noticeable difference between the rice-fish and rice only bay. A snapshot near harvest showed substantially lower levels of weeds present in rice-fish paddies in comparison to rice paddies without fish (Fig 3).

## 4. Discussion

Rice-fish concurrent co-culture has been practiced for centuries in Asia, while in the U.S. only sequential (rice-fish yearly rotations) rice-fish culture has been evaluated. We demonstrated in the present studies for the first time that Koi carp at two different sizes can be co-cultured concurrently in flooded rice paddies and result in positive estimated net returns. While it is unlikely that concurrent rice-Koi co-culture in the U.S. is a viable enterprise on large, industrial-scale rice farms, it appears to offer potential as a diversification strategy for smaller fields where specialty rice varieties are grown. Applicability of this production model would be limited to rice-growing regions in the United States (Arkansas, California, Louisiana, Mississippi, Missouri, and Texas). Th**e** results from our study were promising. However, incorporating fish into a rice production enterprise will require economic and marketing feasibility studies to determine if it can be successful at larger scales, in different geographic locations, and with different fish species and rice varieties. Implementation requires, among other things, additional planning, specialized equipment, personnel training, and modified crop management.

### 4.1. Size of fish

Results of these studies suggest that Koi carp initial weight affected gross fish yield. Based on prior studies from Europe [28–30], during Study 1 planning an expected 400 kg/ha gross Koi yield was assumed whereas only 277 kg/ha were recovered at harvest because of bird predation; a gross Koi yield of 403 kg/ha would be achieved if Study 1 survival was similar to Study 2 (ca. 83%). An expected 600 kg/ha gross Koi yield was assumed during planning Study 2 based on a 150-g initial fish weight. Stocking the larger Koi (ca. 300 g/fish) in Study 2 yielded nearly 890 kg/ha gross fish yield, well within the 850−1,200 kg/ha gross yield reported for carp stocked in concurrent rice-fish culture systems in other studies (28, 29, 30). However, carp were fed a supplemental feed in the cited studies, whereas the Koi carp in Study 2 foraged exclusively on natural food sources.

Fish size was an important factor in the studies. In 2023, many fish were lost during the season due to size differences compared to 2024. Stocking small (ca. 15 g/fish) Koi carp fingerlings resulted in net fish production nearly 25% higher than stocking larger (ca. 300 g/fish) fish. However, predation of the small fingerlings by Great Egrets (*Ardea alba*), despite varied efforts to keep the birds away, contributed to the low survival obtained. On the other hand, fingerling cost and “net” revenue was higher with small fingerlings. The large Koi carp had a higher survival rate but were not immune to attempted predation by Great Egrets. Unlike with small fingerlings, we did not observe Great Egrets consuming the large Koi carp, but various large, dead Koi carp with bird pecks were recovered. Although stocking small Koi carp fingerlings appears to be a more viable approach, management of bird predation will be critical to success. Thus, refinement of Koi carp culture protocols and an economic evaluation still are needed.

### 4.2. Type of fish

Another important consideration for concurrent rice-fish co-culture in the U.S.A. is fish species. Koi carp were selected because of their food habits, environmental tolerance, and economic value as an ornamental fish given their varied, mottled coloration patterns. Fathead minnow (*Pimephales promelas*) is a commercially grown baitfish being evaluated currently for sustainable co-cultivation in a sequential rice-fish rotation [18]. While the fathead minnow, like Koi carp, has broad environmental tolerance and feeds on detritus and algae, they do not feed on plant material [25,36]. Goldfish (*Carassius auratus*), a commercially produced bait and aquarium fish, has food habits and environmental tolerances like those of Koi carp but likely would be more susceptible to bird predation because of its uniform, bright orange coloration [24,25]. Growing food fish, e.g., channel catfish (*Ictalurus punctatus*) may have limited potential because supplemental feeding is required for fast growth.

### 4.3. Weed control

Studies in Asia have documented rice-fish co-culture reduces the need for additional fertilizer by 24% while reducing the release of excess nitrogen into the environment and may decrease pesticide and herbicide use by up to 68% [37,38]. In the present study, we observed that Koi carp controlled many of the weed species present in rice fields. In turn, the Koi provided organic fertilizer through their excrement. In this regard, then larger, more mature Koi might also be somewhat better at controlling weeds than juveniles/fingerlings through both their feeding and digging/rooting behaviors; further investigation is needed. Ultimately, this reduces the need for chemical herbicides and fertilizers, making co-cultivation environmentally benign. In 2023 and 2024, bays with fish had fewer aquatic weeds and algae, indicating that the fish influenced the aquatic weeds and algae. However, in 2023 barnyard grass presented a problem. Barnyard grass is a monocot grass species like rice. In 2023 barnyard grass established a foothold in the rice bays prior to the introduction of fish and as with rice, the fish did not appear to hinder the growth of barnyard grass. In 2024, herbicides were applied to the bays before fish were introduced and were effective at controlling weeds, particularly the barnyard grass, which allowed the rice plots/strips to have good plant stands unlike 2023.

In the current study, there was a significant increase in milling yield and protein content observed with the rice-fish co-cultivation method. Perhaps the biggest benefit of co-cultivation methods was shown in 2024, with a 2x increase of estimated yield after rice was grown together with fish for 4 months. The increase in yield, milling quality, and protein may be due to a range of multiple factors. The nutrient-rich fish excrement may have provided a constant sustainable fertilizer source, particularly during the grain fill stage, and increased nitrogen levels are known to impact protein content, milling yield, and yield in rice [39–41]. In addition, the fish may have decreased competition from weedy species and affected the soil microbiome. The only significant difference in panicle traits or thousand grain weight was a small increase in the number of panicle branches in the rice fish study which was only observed in 2023. Competition from weeds (i.e., the presence of barnyard grass) may have contributed to this difference in 2023. In 2024 barnyard grass in the plots was not a problem and although not significant, primary panicle branches were lower in the rice-fish paddies than rice only paddies.

Another notable benefit of rice-fish co-cultivation was a reduction in weeds. Rice paddies with fish had noticeably fewer weeds than rice paddies without fish, with barnyard grass being the exception in 2023. Weeds compete with rice for space and sunlight while taking up nitrogen and other nutrients from the soil. The fish serve a dual purpose in controlling the weeds while releasing a constant supply of bioavailable fertilizer and micronutrients for the rice plants to support their growth and yields. The fish in the rice paddies were not provided with supplemental food and subsisted on weeds, algae, insects/invertebrates, and other natural organic matter in the paddies, yet still had an impressive 623% increase in weight (g/fish) in 2023 and up to 48% weight (g/fish) gain in 2024. Although not examined in these studies, it is likely the excrement and feeding habits of the fish caused rice-fish paddies to be more nitrogen-efficient and have quicker rates of organic matter decomposition, than the monoculture plots. The cost and sustainability advantages of a free food source for the fish, reduced need for herbicides/pesticides, and increased rice yields from natural fertilizers are a few potential benefits of co-cultivation techniques.

During the last 5 years the gross cost of rice produced per hectare in the U.S.A. has averaged $3534 and the average rice farm size is 242 hectares (https://www.ers.usda.gov/data-products/commodity-costs-and-returns, accessed 5/28/2025) [42]. Specialty rice varieties as well as higher quality rice can both significantly increase profits. Whole milled rice (referred to as head rice) often commands prices 50% higher than broken kernels, so while yield is important, milling quality is also a critical factor in determining the value of the crop. Our studies show that rice-fish co-cultivation significantly increased yield by 129% and milling quality by 58% compared to growing rice alone. For small plots growing higher value specialty rice the potential increase in value from getting more than double the yield plus an increased percentage of whole grains from improved milling quality could be significant. While co-cultivation techniques applied to the entire acreage of an average sized rice farm of 242 hectares is likely unfeasible, co-cultivation techniques on a smaller scale could help both large and small acreage farmers increase the variability of the products they offer and maximize profits from smaller select portions of their land.

Mass producing a large monoculture crop often streamlines efficiency but also leaves the producer vulnerable to swings in commodity prices. With co-cultivation techniques, farmers can produce income from two or more products on the same area of land that may have widely differing market values depending on the year. This can provide a monetary buffer for farmers during years when the market value for one of the products is low. An example of this was seen recently in Louisiana, U.S.A. where co-cultivation of rice and crawfish has been practiced for many years. Most years, rice-crawfish co-cultivation is typically more profitable for crawfish than rice, but during COVID the closure of many restaurants caused demand for crawfish to plumet, and prices declined dramatically while rice prices stayed high. A similar scenario occurred in 2024 with crawfish production being hampered by white spot syndrome virus and weather-related events. Co-cultivation techniques can help increase income options available to farmers while providing protection during commodity fluctuations.

Monocrop fields require heavy applications of fertilizers, pesticides and herbicides to ramp up yields, whereas co-culturing rice and fish may reduce many of these costly and environmentally detrimental applications [43]. To ensure long-term success and sustainability of co-culture systems, it will be imperative to incorporate effective strategies into an overall management plan. These strategies may include regular monitoring, appropriate treatment usage, and crop rotation. The optimum fertilizer/feed, stocking density of fish species, and economic optimum in different rice spacing densities will be investigated in further studies.

#### Future directions.

The idea of co-cultivation techniques is not new. For centuries rice farmers in Asia have practiced co-culturing rice and fish to boost their yields and increase the diversity of food available to eat and sell. However, these methods are giving way to the principles of monoculture, to supply a globalized food system. By examining the interplay between fish and rice plants, this study helps to build the scientific case for why this century-old farming approach is one we should consider in our agricultural ecosystem, particularly in regards for its potential use for small scale specialty rice farmers. Co-cultivation techniques will require changes in culture practices and equipment. For example, a suitable fish holding tank and/or pond will be needed by most farmers wishing to transition to rice-fish co-cultivation methods and additional labor may be required to monitor and harvest fish. In the future, investigation of co-culture of Koi carp, tilapia, or other fish in different rice spacing densities and with different rice varieties with varying growth periods can be examined with the goal to create balanced soil characteristics, primary production, and strategic resource utilization for sustainable rice-fish production systems in the southern U.S.A.

## Conclusions

This study suggests rice-fish co-cultivation in the U.S.A. can be beneficial for farmers. In the present study a high value ornamental fish at two separate size ranges were evaluated with a premium rice variety. In Studies 1 and 2, production of Koi carp generated estimated net returns of $9,264/ha and $7,442/ha. Rice in the rice-fish bays had better grain quality and yield than rice grown without fish. Grain from the rice-fish bay had a 22.6% increase in percent protein, a 58% increase in milling yield, and an impressive yield increase of 129%. In this study rice-fish co-culture appears profitable and provides economic and environmental benefits, particularly when a high value fish species is used in manageable plot sizes. The results of this study, while promising, are not meant to be construed as an exact measurement of expected profits from co-cultivation, but rather the first step in determining if the technique is feasible in the Southern U.S.A. As with any farming endeavor changes in commodity prices, fish mortality, predation, and a host of factors could negatively impact production and input costs would need to be carefully evaluated. Additional studies would need to be performed to assess the utility and potential benefits of rice-fish co-culture with larger plot sizes, varying fish species, and rice varieties. Nevertheless, rice-fish co-culture appears to synergistically generate essential carbohydrates and proteins in a common culture system that increases potential income for food producers.

## Supporting information

S1 Table Chemical composition of trademarked herbicides. (XLSX)

S2 Table 2023 rice panicle traits and 1K grain weight. (XLSX)

S3 Table 2024 rice panicle trait data and 1K grain weight. (XLSX)

S4 Table 2024 milling yield and yield. (XLSX)

S5 Table 2024 protein % of rice grains. (XLSX)

S6 Table Water characteristics. (XLSX)

S7 Table Koi stock out. (XLSX)

S8 Table Koi final weight. (XLSX)

S9 Table Koi harvest. (XLSX)

S10 Table 2023 paddy dissolved oxygen, temperature, and pH. (XLSX)

S11 Table 2024 paddy dissolved oxygen, temperature, and pH. (XLSX)
